# An improved medium for the anaerobic growth of *Paracoccus denitrificans* Pd1222

**DOI:** 10.3389/fmicb.2014.00018

**Published:** 2014-01-31

**Authors:** Stefanie M. Hahnke, Philipp Moosmann, Tobias J. Erb, Marc Strous

**Affiliations:** ^1^Microbial Fitness Group, Max Planck Institute for Marine MicrobiologyBremen, Germany; ^2^Microbial Physiology, Institute of Microbiology, Eidgenössische Technische Hochschule (ETH) ZürichZürich, Switzerland; ^3^Microbiology of Sustainable Energy Production, Center for Biotechnology, Institute for Genome Research and Systems Biology, University of BielefeldBielefeld, Germany; ^4^Department of Geoscience, Energy Bioengineering, University of CalgaryCalgary, AB, Canada

**Keywords:** *Paracoccus denitrificans*, anaerobic growth, cultivation, defined medium, trace elements, copper, chelator

## Abstract

*Paracoccus denitrificans* is a well studied model organism with respect to its aerobic and anaerobic respiratory enzymes. However, until now, the growth medium for this organism has not been optimized for anaerobic growth. In particular, the requirements of *P. denitrificans* for trace elements (TEs) are not well known. In the present study we aimed to improve growth rates of *P. denitrificans* Pd1222 on a defined medium under anoxic conditions. We designed media containing different combinations of TEs at various concentrations, and tested their performance against previously reported media. Our results suggest that growth rate and yield depend on the availability and concentration of TEs in the medium. A chelated TE solution was more suitable than an acidified TE solution. Highest growth rates were achieved with medium comprising the TEs iron, manganese, molybdenum, copper and zinc ranging from 0.1 to 9 μM. On this medium, *P. denitrificans* Pd1222 grew with a generation time of 4.4 h under anoxic conditions and 2.8 h under oxic conditions. Diauxic growth was clearly shown with respect to nitrate and nitrite reduction under anoxic conditions.

## Introduction

The first strain of *Paracoccus denitrificans* (synonym *Micrococcus denitrificans*) was isolated from soil more than one century ago by Beijerinck and Minkman (1910). It was shown to grow aerobically and anaerobically performing complete or partial denitrification. Although *P. denitrificans* is an important model organism to study the electron transfer chain and energy conservation (for review see Stouthamer, 1991), there is still a lack of detailed information about the requirements of this organism for optimal growth. However, cultivation conditions have an important influence onto the physiological phenotype of an organism. Prominent examples for physiological processes that are strongly influenced by cultivation are the aerobic oxidation of methane (Stanley et al., 1983; Prior and Dalton, 1985) and methanol, the fixation of nitrogen gas (Lehman and Roberts, 1991), and the assimilation of CO_2_, which make use of different enzymes (or even pathways) depending on the composition of the growth medium [most notably the presence or absence of vitamins and trace elements (TEs)]. Consequently, it is important to improve cultivation conditions of an organism and prevent its growth inhibition to avoid misinterpretation of the observed phenotypes.

Several approaches have been developed for medium optimization. Approaches based on evolutionary algorithms have been used lately to develop and optimize media for the isolation of novel bacterial strains without making assumptions about the individual components of a medium (Heylen et al., 2006). In contrast, rational approaches rely on the systematic improvement of existing media by focusing on a particular subset of medium components, such as vitamin or TE supplements. In this study, we followed a rational strategy focusing on TE composition to improve the medium for the anaerobic growth of *P. denitrificans* Pd1222 on acetate.

*P. denitrificans* grows aerobically with maximum growth rate at pH 7.6 and at 36°C and can tolerate salt concentrations of at least 3% (Nokhal and Schlegel, 1983). Whereas the suitability of different carbon sources of *P. denitrificans* has been characterized in detail (Nokhal and Schlegel, 1983; Kelly et al., 2006), its requirements for TEs have not been investigated extensively. Different kinds of TEs, as well as large ranges of concentrations have been previously used in different studies (an overview of common TE solutions used for the cultivation of *P. denitrificans* and related organisms is given in Table 1). These solutions have been frequently used by many researchers (Meijer et al., 1979; Stouthamer and Bettenhaussen, 1980; Van Spanning et al., 1990; Moir and Ferguson, 1994; Sears et al., 1997). TEs are essential for the correct function of enzymes, such as those of the respiratory chain; however, at higher concentrations they can impair growth and even be toxic. Here we present results on improvement of anaerobic growth of *P. denitrificans* Pd1222 with focus on TE requirements.

**Table 1:** **Compositions of frequently used trace element solutions in the literature (final concentrations in μM in the medium)**.

**Property and composition of solution**	**References**							
	**Robertson and Kuenen (1983)**	**Strohm et al. (2007)**	**Lawford (1978)**	**Widdel and Pfennig (1981)**	**Widdel and Bak (1992)**	**Chang and Morris (1962)**	**Nokhal and Schlegel (1983)**	**Harms et al. (1985)**
Metal dissolution	EDTA	Acidic	Acidic	Acidic	EDTA	Iron citrate	Iron citrate	EDTA, citric acid
Na_2_-EDTA	342.2				14.0			
Iron (II)	36.0^a^	7.5^b^		7.5^b^	7.5^a^	19.8^a^		20.0^a^
Iron (III)			90.0^b^				4.6^c^	
Manganese (II)	51.1^b^	0.5^b^	50.0^b^	0.5^b^	0.5^b^	4.5^a^	0.2^b^	13.2^a^
Copper (II)	12.6^a^	0.01^b^	5.0^b^	0.1^b^	0.01^b^		0.1^b^	
Molybdenum (VI)	1.8^d^	0.2^e^	10.0^e^	0.2^e^	0.2^e^	728.4^e^	0.1^e^	599.0^e^
Cobalt (II)^b^	13.5	0.8	10.0	0.8	0.8		0.8	
Zinc (II)	27.3^a^	0.5^b^	25.0^b^	0.5^b^	0.5^a^		0.4^a^	
Boron (III)^f^		0.1		1.0	0.5		4.9	
Nickel (II)^b^		0.1		0.1	0.1		0.1	
Relevant organism studied	*P. denitrificans* DSM 413^T^	*P. pantotrophus* DSM 65^T^	*P. denitrificans* ATCC 13543	*Desulfobacter postgatei*	Sulfate-reducing bacteria	*Micrococcus denitrificans*	*P. denitrificans* DSM 413^T^ and other strains	*P. denitrificans* various strains
Comments	Modified after Vishniac and Santer (1957)	According to Widdel (1983)	Modified after Light and Garland (1997)				Modified after Pfennig (1974)	
Reproduced in this study	TE-1, Figure 1A	TE-2, Figure 1B	Not shown	Not shown	Figures 2A,B	n.a.	n.a.	n.a.

*TE, trace element solution; n.a., not applicable*.

a *Elements supplied as sulphates*.

b *Elements supplied as chlorides*.

c *Iron supplied as Fe(III)NH_4_-citrate*.

d *Molybdenum supplied as (NH_4_)_6_Mo_7_O_24_*.

e *Molybdenum supplied as Na_2_MoO_4_*.

f *Boron supplied as H_3_BO_3_*.

## Materials and methods

### Organisms

*Paracoccus denitrificans* Pd1222 (16S rRNA gene accession number NR_074152), a derivative of DSM 413^*T*^ (De Vries et al., 1989), was obtained from Prof. Dr. R. van Spanning, Vrije Universiteit Amsterdam, faculty of Earth and Life Sciences. The organism was maintained aerobically on solid LB medium, containing 15 g/L agar, and transferred every three months. For long-term storage the cells were frozen at −80°C in 30% glycerol and revived by spreading frozen cells on LB agar plates and incubating aerobically at 30°C for 2–3 days.

### Mineral salt media

The following chemicals were purchased from AppliChem, Darmstadt, Germany: MgSO_4_ ·7 H_2_O, CaCl_2_ · 2 H_2_O, K_2_HPO_4_ and sodium acetate. All other chemicals were received from Carl Roth GmbH, Karlsruhe, Germany. The purity was at least 99% for most chemicals, except for ZnCl2, 97%, MnCl_2_ · 4 H_2_O, 98% and NiCl_2_ · 6 H_2_O, 98%, whereas major impurities comprised sulphates or chlorides.

The experiments presented here were grouped into series 1–5. The mineral salt medium of series 1 was prepared after Taylor and Hoare (1969). The medium was supplemented with two different TE solutions which are described below. For series 2–5, a freshwater medium modified after Widdel and Bak (1992) was used, containing (in g/L): NH_4_Cl (0.5), MgSO_4_ · 7 H_2_O (0.5), CaCl_2_ · 2 H_2_O (0.1), KH_2_PO_4_ (0.04), K_2_HPO_4_ (0.12), and HEPES (6.0). Phosphate was added from a separately prepared and autoclaved stock solution. This medium was used to test three different TE solutions previously described (see below).

Materials used for medium preparation were rinsed with ultra pure water (Aquintus system, membraPure, Germany) prior to usage. All media were prepared with ultra pure water and the pH was adjusted to 7.2 with 1 M HCl or 1 M NaOH if necessary. The media were supplemented with 60 mM sodium acetate as carbon and energy source and 30 mM KNO_3_ as the electron acceptor were added to both anaerobic and aerobic cultures to ensure identical salt concentrations, unless otherwise stated. For anaerobic cultures, 10 mL or 30 mL mineral salt solutions including electron donor and acceptor were filled into Hungate tubes or 50 mL serum flasks using a volumetric pipette, and capped with a butyl stopper. The headspace was exchanged by applying vacuum, supplying argon at a pressure of 1.5 bar, followed by rigorous shaking (Widdel and Bak, 1992). This procedure was repeated three times. Finally, overpressure was released through a second needle. For aerobic cultures, 50 mL medium were filled into 250 mL Erlenmeyer flasks. All media and stock solutions were autoclaved at 121°C for 25 min.

### Trace element solutions

The final TE concentrations in the media for all growth experiments in this study are listed in Tables 1 and 2. Five TE stock solutions that have been previously presented, were prepared, three acidic solutions and two solutions containing a chelator (Table 1). Solution TE-1 was prepared according to Vishniac and Santer (1957) as described by Robertson and Kuenen (1983) and contained (in mg/L): Na_2_-EDTA (5000), FeSO_4_ · 7 H_2_O (500), CaCl_2_ · 2 H_2_O (728), MnCl_2_ · 4 H_2_O (506), CuCl_2_ · 2 H_2_O (107), Na_2_MoO_4_ · 2 H_2_O (22), CoCl_2_ · 6 H_2_O (161), and ZnCl_2_ (185). The acidic solution TE-2 was prepared according to Widdel (1983). The medium after Taylor and Hoare was supplemented with 2 mL/L solution TE-1, a combination which was previously used for the cultivation of *P. denitrificans* DSM 413^*T*^ (Robertson and Kuenen, 1992). Alternative growth experiments were performed with the same medium amended with 1 mL/L solution TE-2. Three more TE solutions were prepared (Lawford, 1978; Widdel and Pfennig, 1981; Widdel and Bak, 1992) and tested with medium after Widdel and Bak (1992). The solution after Lawford was previously used for the cultivation of *P. denitrificans* ATCC 13543 (Lawford, 1978). Series 2 of the growth curves was performed with a set of TE solutions, designed in this study (Table 2), with increasing Cu^2+^ concentrations that contained (in mg/L): Na_2_-EDTA (7300), FeSO_4_ · 7 H_2_O (2500), MnCl_2_ · 4 H_2_O (20), Na_2_MoO_4_ · 2 H_2_O (242), CuCl_2_ · 2 H_2_O (17; 38; 85; 128; 170; 426). TE solutions for the growth experiments of series 3–5 (Table 2) comprised the components of the solutions of series 2 (with 85 mg/L CuCl_2_ · 2 H_2_O) and optional (in mg/L): CoCl_2_ · 6 H_2_O (238), ZnCl_2_ (340), H_3_BO_3_ (30), NiCl_2_ · 6 H_2_O (24). Depending on the final TE concentrations, the errors caused by impurities of chemicals were: 0.7–7.4% (iron), 0.5–4.6% (Co), 0.2–8.4% (Zn), 0.7–3.6% (Mn), and 0.1–2.6% (Mo). The impact of impurities of copper and nickel was higher; therefore the concentrations given in the results were corrected for this error.

**Table 2:** **Compositions of trace element solutions tested in different series of growth experiments in this study (final concentrations in μM in the medium)**.

**Compound**	**Series 1**		**Series 2^c^**	**Series 3^d^**						**Series 4^e^**	**Series 5^f^**
	**TE-1^a^**	**TE-2^b^**		**TE-3**	**TE-3-Co**	**TE-3-Zn**	**TE-3-B**	**TE-3-Ni**	**TE-4**	**TE-3-Zn**	**TE-3-Zn**
EDTA	342.2		19.6	19.6	19.6	19.6	19.6	19.6	19.6	19.6	19.6
Iron (II)	36.0	7.5	9.0	9.0	9.0	9.0	9.0	9.0	9.0	9.0	9.0
Manganese (II)	51.1	0.5	0.1	0.1	0.1	0.1	0.1	0.1	0.1	0.1	0.1
Copper (II)	12.6	0.01	0.4 − 2.8	0.8	0.8	0.8	0.8	0.8	0.8	0.8	0.8
Molybdenum	1.8	0.2	1.0	1.0	1.0	1.0	1.0	1.0	1.0	1.0	1.0
Cobalt (II)	13.5	0.8			1.0				1.0		
Zinc (II)	27.3	0.5				2.5			2.5	2.5	2.5
Boron		0.1					0.5		0.5		
Nickel (II)		0.1						0.07	0.07		
pH	6	1	6	6	6	6	6	6	6	6	6

a Trace element solution after Vishniac and Santer (1957)

b *Trace element solution after Widdel (1983)*.

c *Test of the impact of copper on growth*.

d *Test of the impact of other trace elements on growth (also tested at ten times higher concentrations)*.

e *Aerobic growth on the improved medium*.

f *Temperature dependence of anaerobic growth on the improved medium*.

TE solution TE-1 and all solutions of series 2–5 were prepared as chelated stock solutions and the pH was adjusted to 6 with 5 M NaOH. Stock solutions were autoclaved at 121°C for 25 min and added to the media with syringes. To reduce the volumetric error, the TE stock solutions were diluted ten or one hundred times, allowing the addition of a larger volume to the mineral media in Hungate tubes.

### Anaerobic and aerobic growth experiments

Depending on the medium to be tested, the inoculum was prepared in different ways: (1) several colonies from solid LB medium were suspended in 1 mL of the respective mineral salt solution described above. (2) Cells were grown aerobically on the medium to be tested to a final optical density (OD_600_) of 0.2. Both aerobic and anaerobic cultures were inoculated with 1% cell suspension. The cultures were incubated at 30°C. Aerobic cultures were shaken constantly at 120 rpm. Anoxic cultivation at different temperatures (11–45°C) was performed in Hungate tubes in a temperature gradient metal incubator.

### Sample withdrawing and analyses

From cultures with an initial volume of 30 mL or more, samples of 1–1.5 mL were taken aseptically in different time intervals depending on medium and growth conditions. Bacterial growth was followed by measuring the OD_600_ at 600 nm (cuvette path length 1 cm) with a spectrophotometer (Genesis 10S UV-VIS, Thermo Scientific). Three replicate growth curves were additionally followed by protein determination (Lowry et al., 1951), revealing a linear relationship between OD_600_ and protein concentration. Subsequently, the sample was centrifuged at 14500× *g* at 4°C for 5 min. The supernatant was removed and stored at −20°C for nitrate, nitrite and acetate determination. The cultures in Hungate tubes were not sampled. Instead, the optical density was measured at 600 nm directly in the culture tube with a spectrophotometer (UV-1201V, Shimadzu) in different time intervals depending on medium and growth conditions. The OD_600_ values were corrected corresponding to measurements in cuvettes with 1 cm path length to allow comparison between all measured OD_600_ values.

Nitrate was determined colorimetrically after reaction with salicylic acid (Cataldo et al., 1975). Nitrite was determined colorimetrically using the Griess-Romijn reagent (Griess-Romijn-van Eck, 1966). Acetate was determined with an HPLC system (Sykam GmbH) at a retention time of 13.8 min and a detection limit of 25 μM acetate. With 5 mM H_2_SO_4_ as eluent and a flow rate of 0.6 ml min^−1^, acetate was detected using a refractive index (RI) or UV detector (210 nm).

## Results

**Figure 1:**
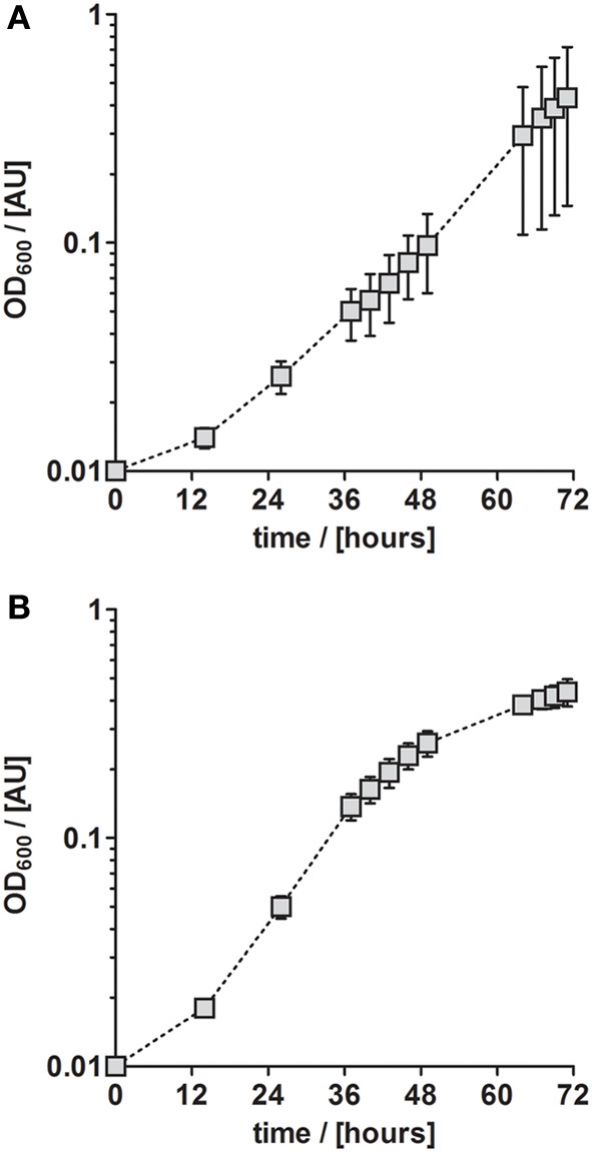
**Anaerobic growth of *P. denitrificans* Pd1222 on published media**. Growth was performed on two media previously described for *P. denitrificans* or *P. pantotropha* with trace element concentrations as given in Table 1. **(A)** Growth with chelated trace element solution TE-1 after Robertson and Kuenen (1983). **(B)** Growth with acidic trace element solution TE-2 after Strohm et al. (2007). The graphs represent three replicates; error bars indicate standard deviation and may be smaller than the symbols.

Although *P. denitrificans* is a model organism for denitrification, we found that the growth media and TE solutions described for this organism enabled only very poor anaerobic growth of *P. denitrificans* Pd1222. This phenomenon was most apparent when growing this strain on medium (Taylor and Hoare, 1969) that was amended with either TE solution TE-1 (Vishniac and Santer, 1957; Robertson and Kuenen, 1983) or TE-2 (Widdel, 1983; Strohm et al., 2007). Both TE solutions have been used before for the cultivation of *P. denitrificans* or *P. pantotrophus* and are listed as final concentrations in the medium in Table 1. TE-1 is characterized by high metal concentrations between 1.8 and 36 μM and contains ethylenediaminetetraacetic acid (EDTA) as a chelator to keep the metals dissolved, while metals in TE-2 are 4 to more than 1000 times lower concentrated and metal dissolution is achieved by lowering the pH instead of adding EDTA. Under anoxic, denitrifying conditions, very slow growth was detected with a generation time of 12.3 h in medium supplemented with TE-1 and 9.3 h in medium supplemented with TE-2 (Figure 1; for specific growth rates, please also refer to Table 3). Thus, we sought for a defined medium which was more suitable for the cultivation of *P. denitrificans* Pd1222 under anoxic, denitrifying conditions.

**Table 3:** **Growth characteristics of *Paracoccus denitrificans* Pd1222, grown in batch cultures under anoxic, denitrifying conditions unless otherwise stated**.

**Growth experiment**	**Parameter**	**Growth μ (h^−1^)**	**Generation time (h)**
**TRACE ELEMENT SOLUTION**			
Medium according to the literature (Figure 1)	TE-1	0.056 ± 0.013	12.3
	TE-2	0.075 ± 0.002	9.3
**ACETATE CONCENTRATION (mM)**			
Trace elements according to Widdel and Bak (1992); increasing substrate concentrations (Figure 2)	10	0.086 ± 0.002	8.1
	20	0.092 ± 0.011	7.6
	40	0.093 ± 0.001	7.4
	60	0.095 ± 0.004	7.1
	100	0.097 ± 0.006	7.6
**Cu^2+^ CONCENTRATION (μM)**			
Increasing Cu^2+^ concentrations, anaerobic	0.4	0.123 ± 0.004	5.7
	0.5	0.134 ± 0.026	5.2
	0.8	0.133 ± 0.012	5.2
	1.0	0.129 ± 0.044	5.4
	1.3	0.157 ± 0.003	4.4
	2.8	0.144 ± 0.010	4.8
Increasing Cu^2+^ concentrations, aerobic	0.5	0.159 ± 0.008	4.4
	0.8	0.089 ± 0.008	7.8
	1.0	0.054 ± 0.002	12.8
	1.3	0.029 ± 0.003	18.6
	2.8	0.021 ± 0.007	32.4
**TRACE ELEMENT SOLUTION**			
Different trace element compositions (Figure 3A)	TE-3	0.129 ± 0.002	5.4
	TE-3-Co	0.154 ± 0.011	5.2
	TE-3-Zn	0.159 ± 0.002	4.4
	TE-3-B	0.135 ± 0.004	5.1
	TE-3-Ni	0.141 ± 0.017	4.9
	TE-4	0.168 ± 0.001	4.7
10× concentrated trace elements (Figure 3B)	TE-3	No growth (<0.008)	No growth (>84 h)
	TE-3-Co	0.104 ± 0.007	6.7
	TE-3-Zn	0.125 ± 0.033	5.5
	TE-3-B	No growth (<0.008)	No growth (>84 h)
	TE-3-Ni	No growth (<0.008)	No growth (>84 h)
	TE-4	0.129 ± 0.035	5.4
Aerobic growth (Figure 4)	TE-3-Zn	0.251 ± 0.032	2.8
**TEMPERATURE (°C)**			
Increasing temperature (Figure 5)	11.2	0.010 ± 0.001	67.7
	16.1	0.023 ± 0.001	30.3
	19.3	0.044 ± 0.001	15.9
	21.0	0.056 ± 0.002	12.5
	22.5	0.063 ± 0.003	11.0
	24.2	0.066 ± 0.004	10.4
	25.9	0.061 ± 0.008	11.3
	27.4	0.084 ± 0.006	8.3
	29.0	0.093 ± 0.009	7.5
	30.5	0.104 ± 0.004	6.7
	32.1	0.152 ± 0.004	4.6
	33.7	0.188 ± 0.007	3.7
	35.3	0.201 ± 0.008	3.4
	36.9	0.214 ± 0.015	3.3
	40.0	0.161 ± 0.008	4.3

**Figure 2:**
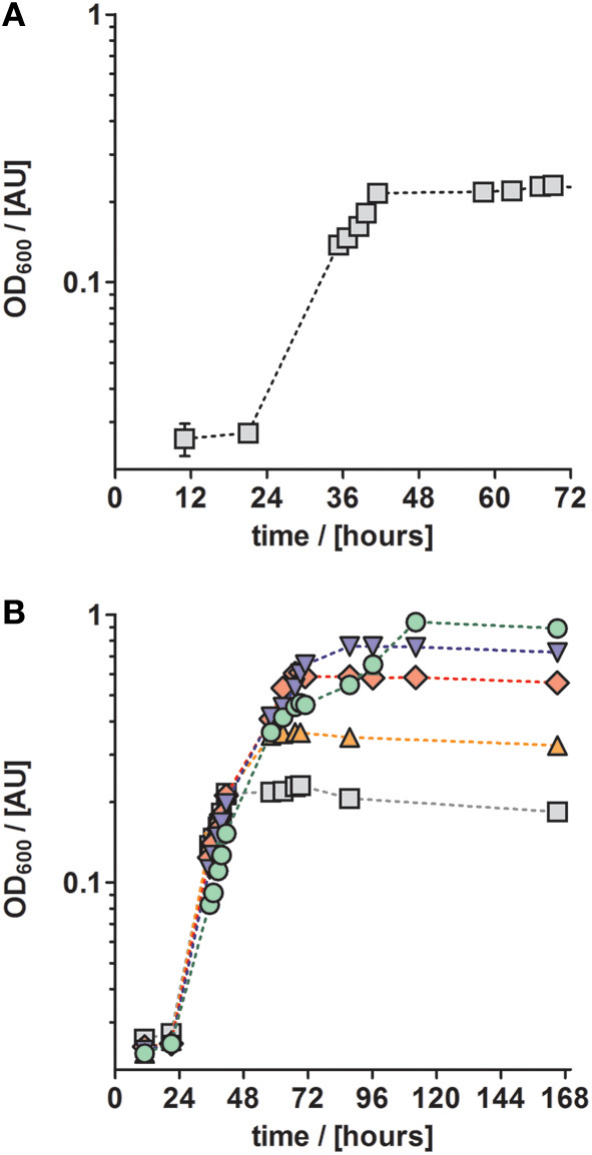
**Growth curves of anaerobically grown *P. denitrificans* Pd1222 on freshwater medium supplemented with a chelated trace element solution after Widdel and Bak (1992, Table 1). (A)** Growth with 5 mM nitrate and 10 mM acetate. **(B)** Growth with different concentrations of electron donor (acetate) and acceptor (nitrate) to investigate whether a component of the medium is limiting. Concentrations were 5 mM nitrate, 10 mM acetate (gray squares), 10 mM nitrate, 20 mM acetate (orange up triangles), 20 mM nitrate, 40 mM acetate (red diamonds), 30 mM nitrate, 60 mM acetate (blue down triangles), 50 mM nitrate, 100 mM acetate (green circles). The graphs represent three replicates; error bars indicate standard deviation and may be smaller than the symbols.

Anaerobic growth of *P. denitrificans* Pd1222 was investigated with a different freshwater medium that was modified after Widdel and Bak (1992) and three different TE compositions as previously described (Lawford, 1978; Widdel and Pfennig, 1981; Widdel and Bak, 1992). The acidic TE solution after Lawford (Table 1) contained up to 100 times higher metal concentrations than the two other TE solutions (Table 1) that were prepared either as an acidic or a chelated (EDTA containing) solution. All media supplemented with acidic TE solutions supported only limited growth under anoxic, denitrifying conditions after one week of incubation. In contrast, good growth was observed in the medium supplemented with chelated TE solution according to Widdel and Bak (1992), with a generation time of 8.1 h (Figure 2A and Table 3, at 10 mM acetate). This indicated that at least one essential compound was insufficiently available to the cells in the absence of a chelator. Based on these results all subsequent growth experiments were performed with TE solutions that contained EDTA for dissolution. The suitability of media subsequently tested, was estimated based on growth rates that are summarized in Table 3, and described in more detail below.

Trace metals such as iron and copper are required for the biosynthesis of essential enzymes, such as the membrane bound protein complexes of the aerobic and anaerobic respiratory chain. Thus we investigated whether the absence of an essential TE might have been the reason for growth limitation. For this purpose, we cultured *P. denitrificans* Pd1222 with increasing acetate and nitrate concentrations in the medium. In the absence of any (trace) element limitation, higher substrate concentrations should lead to proportionally higher growth yields. Figure 2B shows the growth curves of cultures supplied with 10–100 mM acetate (5–50 mM nitrate). With 10 mM acetate and 5 mM nitrate the maximum optical density (maxOD_600_) reached 0.23, whereas at 20 mM acetate and 10 mM nitrate the maxOD_600_ reached 0.37, which is only a 1.6-fold increase instead of the expected 2-fold increase in maxOD_600_. At ten times higher acetate and nitrate concentrations, the effect was even more pronounced, as the maxOD_600_ was 0.96, which corresponded only a 4-fold increase instead of the 10-fold maxOD_600_ which would be expected in the absence of any (trace) element limitation. In short, the increase in OD_600_ was never proportional to the increase of substrate concentration. We may explain this observation in two ways. First, the limited availability of at least one TE might have limited growth. Second, non-optimal growth conditions might have promoted the accumulation of toxic metabolic intermediates that inhibit growth. In this case, the organism would require additional energy as biochemical response to the stress conditions at the cost of growth yield. The latter could also be caused by an inappropriate TE composition, leading to the accumulation of toxic intermediates (Sullivan et al., 2013). Thus, we examined whether growth rates could be improved by providing different concentrations of TEs.

Copper was the first TE that was investigated in more detail. It was supplied to different batch cultures in concentrations from 0.4 to 2.8 μM. In this series we used a TE solution containing (besides copper) only iron, manganese, and molybdenum, as these elements were sufficient to sustain aerobic and anaerobic growth of *P. denitrificans* (Chang and Morris, 1962). Our results did not suggest any trend in generation times with increasing copper concentration (between 4.4 and 5.7 h, Table 3). However the reproducibility was better at lower copper concentrations; at higher copper concentrations (1.0 μM and higher) at least one out of three replicate cultures did not grow. Overall a copper concentration of 0.8 μM was shown to be high enough for growth and low enough to prevent observed negative effects on growth. For comparison, we grew *P*. *denitrificans* Pd1222 with increasing copper concentrations under oxic conditions (Table 3). In this case, generation times increased with increasing copper concentrations, with a minimum of 4.4 h in medium supplemented with 0.4 μM copper and a generation time of 7.8 h at 0.8 μM copper. Growth was severely affected above 1.0 μM copper (generation time 12.8 h). Thus, a copper concentration of 0.8 μM was provided in the minimal TE solution TE-3 (Table 2) that was used for subsequent cultivation of *P*. *denitrificans* Pd1222 under anoxic conditions.

**Figure 3:**
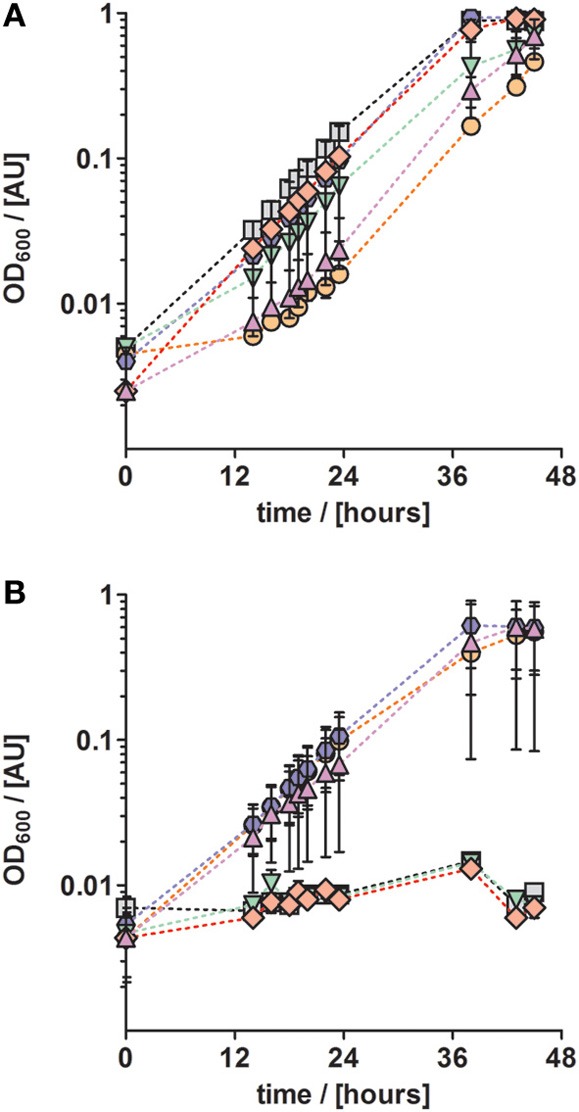
**Anaerobic growth of *P. denitrificans* with respect to different trace element compositions and concentrations**. The influence of a set of trace elements on growth was studied by growing *P. denitrificans* with six different trace element compositions, as listed in Table 2 (series 3). **(A)** Solution TE-3 (purple up triangles); TE-3-Co, solution TE-3 with additional cobalt (orange circles); TE-3-Zn, solution TE-3 with additional zinc (blue hexagons), TE-3-B, solution TE-3 with additional borate (green down triangles); TE-3-Ni, solution TE-3 with additional nickel (red diamonds); TE-4, solution TE-3 containing all additional elements (gray squares). **(B)** The same trace element solutions as described in (A) were supplied at ten times higher concentrations. Growth curves depicted in all panels represent at least two duplicates; error bars indicate standard deviation and may be smaller than the symbols.

Next, we investigated the effect of other TEs on growth. For that purpose, TE solution TE-3 that contained only the four essential elements as described above, was amended with one of four additional TEs (cobalt, zinc, boron, and nickel) to obtain four modified TE-3 solutions as listed in Table 2. Solution TE-4 was obtained by adding all four TEs to solution TE-3. Growth of *P. denitrificans* Pd1222 was examined at two different concentrations of each modified TE-3 solution and TE-4. *P. denitrificans* Pd1222 grew in all cultures containing low concentrations of TEs (Tables 2 and 3; Figure 3A). In medium supplied with TE-3 the cultures grew at a generation time of 5.4 h. The shortest generation time of 4.4 h was achieved with the addition of TE-3-Zn, indicating that zinc positively affected growth of *P. denitrificans* Pd1222. Cultures supplemented with TE-3-Co and TE-3-B resulted in growth with a prolonged lag phase of approximately 22 h (10 h longer than in all other treatments). When combining all individual tested TEs into one TE solution TE-4, medium complemented with TE-4 enabled growth at a generation time of 4.7 h and did not show any improvement over the medium complemented with TE-3-Zn.

In cultures supplied with ten times higher concentrations of TE-3, modified TE-3 solutions and TE-4 solution growth was impaired as shown in Figure 3B (note that most of the final metal concentrations in these media correspond to concentrations that were reached with the TE solution after Lawford). No growth was observed in cultures supplied with ten times concentrated TE-3, TE-3-B, or TE-3-Ni after one week of incubation, and only two out of three replicate cultures grew on medium supplied with ten times concentrated TE-3-Co, TE-3-Zn, or TE-4. In this case, generation times were generally higher than in medium containing low concentrations of TEs as given in Table 2.

**Figure 4:**
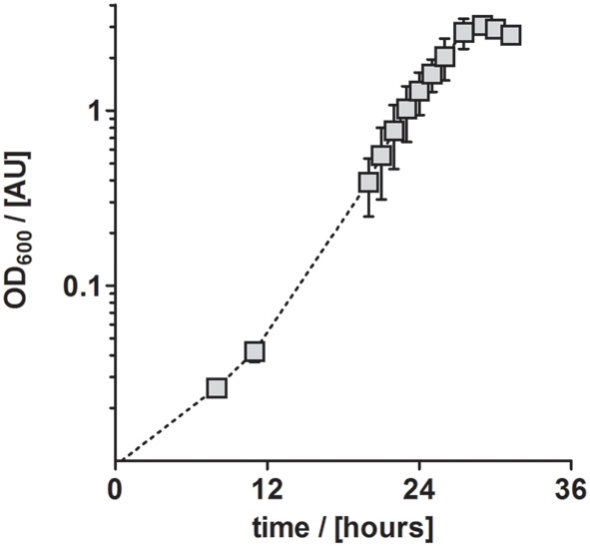
**Aerobic growth of *P. denitrificans* Pd1222 on the improved medium**. Growth was performed with trace element solution TE-3-Zn with concentrations as given in Table 2 (series 4). The graphs represent three replicates; error bars indicate standard deviation and may be smaller than the symbols.

In conclusion, TE solution TE-3-Zn was the most suitable TE solution among all solutions tested in this study for the anaerobic, denitrifying growth of *P. denitrificans* Pd1222. With this solution, a highly reproducible and short generation time of 4.4 h was achieved. Compared to aerobic growth with a generation time of 2.8 h (Figure 4), the generation time was only 1.6 times longer (Table 3).

**Figure 5:**
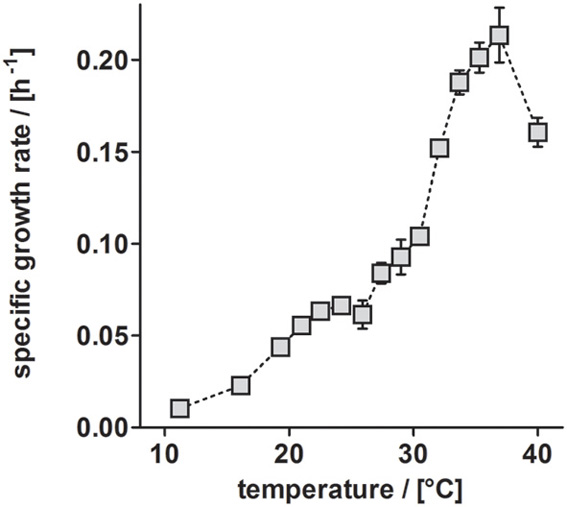
**Dependence of anaerobic growth rates of *P. denitrificans* Pd1222 on temperature**. The growth rates were determined from the early exponential growth phases of duplicate or triplicate growth curve measurements on the improved medium, supplemented with trace element solution TE-3-Zn (Table 2, series 5). Error bars indicate standard deviation and may be smaller than the symbols.

Besides TEs, temperature has a substantial influence on growth (Table 3). Anaerobic growth of *P*. *denitrificans* Pd1222 in medium containing TE solution TE-3-Zn was observed between 11 and 40°C (Figure 5), with maximum growth rates at 37°C, similar to what was reported by Nokhal and Schlegel (1983). The generation time was 3.2 h. As compared to previous experiments in this study, these were the highest growth rates observed for anaerobic denitrifying growth of *P. denitrificans* Pd1222.

**Figure 6:**
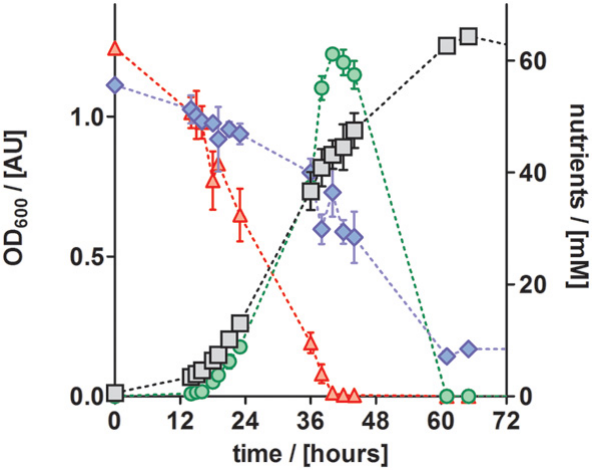
**Growth curve and nutrient analyses of anaerobically grown *P. denitrificans* Pd1222 on the improved medium, supplemented with trace element solution TE-3-Zn (Table 2)**. Growth was followed by OD_600_ measurements (gray squares). Consumption of acetate (blue diamonds) and nitrate (red up triangles), as well as production and consumption of nitrite (green circles) were quantified. All data represent triplicates; error bars indicate standard deviation and may be smaller than the symbols.

To study the performance of *P. denitrificans* Pd1222 during growth on the improved medium in more detail, the conversion of substrates and production of intermediates were analyzed (Figure 6). Nitrate was completely converted to nitrite (the first intermediate in denitrification) in the first half of the exponential growth and nitrite reached a maximum concentration of 62 mM, which corresponded to the initial nitrate concentration. At this point, growth was slowed down, which can be explained by the time required by *P. denitrificans* Pd1222 to induce the expression of nitrite reductase. After approximately 2 h, the growth rate was recovered and nitrite was reduced within 21 h. Accordingly, acetate was consumed with a lower rate during the transition from nitrate to nitrite respiration. This diauxic growth was previously observed (Blaszczyk, 1993; Saéz et al., 2003), but lower concentrations of nitrite were accumulated and consumed than in this study. The depletion of both nitrate and nitrite confirms that the electron acceptor limited growth, implying that trace and macro element concentrations were sufficiently high.

## Discussion

This study aimed at improving cultivation conditions for anaerobic, denitrifying growth of *P. denitrificans* Pd1222, to achieve high growth rates on a minimal medium that are comparable to growth rates in the presence of oxygen. Although *P. denitrificans* has been a long-standing model organism for the study of respiratory enzymes and energy conservation, it was surprising that *P. denitrificans* Pd1222 grew poorly on published media. Thus we sought to test and improve different media that had been used in the past to cultivate this and closely related organisms (Lawford, 1978; Robertson and Kuenen, 1983; Strohm et al., 2007).

Supplementation of media with TEs (and vitamins) in suitable concentrations is known to have an important influence on the successful cultivation of microorganisms (Widdel, 1980; Peters et al., 2004; Pol et al., 2014). Consequently, this study focused on improving the composition of the TE solution. Our experiments suggested that for anaerobic growth of *P. denitrificans* Pd1222, chelator-based TE solutions are superior to solutions in which trace metals are solubilized and kept in their redox state by lowering the pH. Media that were used by many researchers to study *P. denitrificans* contain citrate as chelator (Chang and Morris, 1962; Lawford, 1978; Nokhal and Schlegel, 1983). Since *P. denitrificans* DSM 413^*T*^ has been reported to be able to utilize citrate under oxic, but not under anoxic conditions (Davis et al., 1970; Robertson and Kuenen, 1983), we note that citrate might become a potential carbon source under anoxic conditions, particularly during long-time cultivations. Similar observations have been recently made with *E. coli* (Blount et al., 2008, 2012). Therefore, we used EDTA instead of citrate as the chelator. One previously published medium for *P. denitrificans* DSM 413^*T*^ (Robertson and Kuenen, 1983) also featured EDTA, but in this medium the concentrations of TEs ranged between 13 and 51 μM, notably higher than the 0.1–1 μM which are usually used for TEs (Overmann, 2010, 2013). Higher concentrations of TEs are known to exert toxic effects (Overmann, 2010), and our experiments indeed indicated growth-inhibiting effects of higher concentrations of TEs.

We achieved good growth of *P. denitrificans* Pd1222 with the TEs molybdenum, manganese and copper in concentrations between 0.1 and 1 μM, zinc at 2.5 μM and iron(II) at 9 μM. Three of these elements represent metals required as cofactors by the enzymes of the denitrification pathway of *P. denitrificans* (Stouthamer, 1991). Iron is present in all four respiratory enzymes as Fe-S clusters or as part of a cytochrome domain. The membrane-bound nitrate reductase (Nar) additionally requires molybdenum (Stouthamer, 1991) and the nitrous oxide reductase requires copper (Snyder and Hollocher, 1987). Zinc is not essential for the biosynthesis of the enzymes involved in denitrification, but the increased growth rates in presence of zinc might indicate that it stimulates anaerobic growth in a different way.

In summary, we provide a minimal medium that allows anaerobic growth of *P. denitrificans* Pd1222 at a minimum generation time of 4.4 h at 30°C without the need of vitamin addition. The generation times under anoxic conditions were only 1.6 times longer than under oxic conditions. This may suggest that a more intense study of concentration ranges of all TEs would only result in minor improvements of growth. Our medium is easy to prepare and allows physiological studies of the model organism *P. denitrificans* Pd1222 under anoxic conditions. With this medium, we show that *P. denitrificans* Pd1222 strictly grows diauxically with respect to nitrate and nitrite respiration. Nitrate was completely converted to nitrite before nitrite was reduced to dinitrogen.

We expect that our findings are also relevant to other strains of *P. denitrificans* since previous physiological studies of various strains of *P. denitrificans* showed identical physiological characteristics and similar carbon utilization patterns among the tested strains (Nokhal and Schlegel, 1983). These features specifically relate to major metabolic pathways that constitute the most trace metal dependent enzymes. Therefore, it is likely that the TE composition suggested here represents a suitable composition for more strains of *P. denitrificans*.

## Author contributions

Stefanie M. Hahnke, Tobias J. Erb, and Marc Strous conceived the project, Stefanie M. Hahnke and Philipp Moosmann designed and performed the experiments, Stefanie M. Hahnke and Tobias J. Erb analyzed the data, Stefanie M. Hahnke, Tobias J. Erb, and Marc Strous wrote and edited the manuscript.

### Conflict of interest statement

The authors declare that the research was conducted in the absence of any commercial or financial relationships that could be construed as a potential conflict of interest.
